# Climate Gradients Underlie Geographical Variations in iWUE and δ ^15^N Values of *Encelia*

**DOI:** 10.1002/pei3.70080

**Published:** 2025-08-28

**Authors:** Tegan E. Lengyel, Iman Karavan‐Jahromi, Avery W. Driscoll, James R. Ehleringer

**Affiliations:** ^1^ School of Biological Sciences University of Utah Salt Lake City Utah USA; ^2^ Department of Soil and Crop Sciences Colorado State University Fort Collins Colorado USA

**Keywords:** *Encelia*, intrinsic water use efficiency, isoscape, Mojave Desert, Sonoran Desert, stable isotopes

## Abstract

This study assessed variations in leaf intrinsic water use efficiency (iWUE) and δ^15^N values among *Encelia*, a genus of drought‐deciduous shrubs distributed across arid regions of southwestern North America between 1972 and 1980 when climates were cooler than today. We hypothesized that geographical variations in climate would significantly influence iWUE, a response to water‐related climate constraints, and δ^15^N values, a proxy for the balance between N_2_ fixation and denitrification. Leaf samples were collected from six species of *Encelia* across 78 sites representing the genus range. The δ^15^N and δ^13^C values of these samples were measured and analyzed to identify drivers of spatial variability. Significant variations among iWUE and δ^15^N values were observed as a function of climate, along a spring–summer precipitation gradient. Precipitation and vapor pressure deficit (VPD) were significant drivers of variations in iWUE values, with iWUE increasing with VPD and/or decreasing precipitation, as would be predicted based on water‐related constraints on leaf gas exchange. Climate values were significant drivers of variations in δ^15^N values, with lower δ^15^N values occurring in cooler temperature, spring‐growing plants (northern latitudes) than in warmer summer‐growing plants (southern latitudes). *Encelia* leaf iWUE and δ^15^N observations suggest few, if any, species‐specific differences; but more likely that there is high plasticity in these values driven by variations in climate.

## Graphical abstract

Variations in the intrinsic water use efficiency and nitrogen isotope ratios of the desert shrub *Encelia* are associated with climate. Higher intrinsic water use efficiencies are associated with drier climates, whereas higher nitrogen isotope ratios are associated with warmer year‐round temperatures.

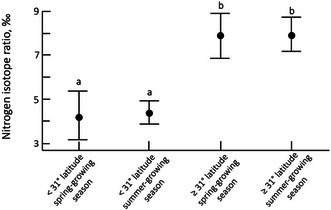

## 1 Introduction

Evaluating spatial patterns in plant stable isotope characteristics allows us to understand how specific species or ecotypes respond to climate conditions today. Such an approach may yield insights into past patterns as well as how a changing climate may influence future distribution patterns and help us understand the extent of ecotypic variation or phenotypic plasticity. This is particularly relevant in desert ecosystems, where limited water and soil nitrogen availability already constrain productivity and affect plant distributions (Chapin III et al. 2002; Schlesinger and Bernhardt 2020). Geographically provenanced stable isotope analyses have been shown to provide information about variations in ecophysiological characteristics (Dawson et al. 2002; Bowling et al. 2008; Bowen 2010; Diefendorf et al. 2010; Cernusak et al. 2013; Cornwell et al. 2018; Adams et al. 2019) and have been used widely to create “isoscapes” (Bowen 2010; West et al. 2010). A well‐established theory quantifies the relationships between leaf carbon isotope ratios (δ^13^C) of C_3_ plants as recorders of intrinsic water‐use efficiency (iWUE) (Farquhar et al. 1989; Ehleringer et al. 1993; Cernusak et al. 2013). Although less rigorously quantified from a theoretical perspective, it is widely accepted that leaf nitrogen (δ^15^N) isotope ratios serve as reliable indicators of the balance between microbial nitrogen fixation and denitrification processes in soils and, therefore, as a measure of soil nitrogen sources (Evans 2001; Amundson et al. 2003; Craine et al. 2009).

Prior studies have successfully integrated δ^13^C and δ^15^N data with climatological data to detect emerging global patterns by synthesizing observations from a range of plant species from across different biomes (Stewart et al. 1995; Miller et al. 2001; Kaplan et al. 2002; Craine et al. 2009; Ordonez et al. 2009; Prentice et al. 2011; Ma et al. 2012). Precipitation has emerged as the likely driver of δ^13^C variations in these synthesis studies. At the experimental level, common garden studies investigating population‐scale and ecotypic variations within a species indirectly support the robustness of water‐related parameters as significant correlates of δ^13^C variations. Ecotypes or populations of a species originating from drier regions of their geographical distribution tended to retain higher δ^13^C values when they were cultivated under common garden conditions (Comstock and Ehleringer 1992; Aitken et al. 1995; Anderson et al. 1996; Guy and Holowachuk 2001; Zhang et al. 2005; Bonhomme et al. 2008; Zhu et al. 2010). In each of the species evaluated in those studies, those populations/ecotypes with higher δ^13^C values also tended to exhibit more conservative δ^13^C values when experimental droughts were imposed in common garden treatments.

Leaf δ^13^C values in C_3_ plants relate to the ratio of intercellular to ambient CO_2_ concentration (*c*_*i*_ */c*_*a*_), thus reflecting discrimination against ^13^C during photosynthesis (Farquhar et al. 1989). Intrinsic water‐use efficiency (iWUE), defined as the ratio of photosynthesis to leaf conductance (*A/g*; Farquhar et al. 1989; Ehleringer et al. 1993), is directly proportional to *c*_*i*_ */c*_*a*_. Thus, iWUE serves as a quantitative measure of plant carbon–water tradeoffs. Understanding those climate‐related factors influencing iWUE can contribute to interpreting the ecological and physiological responses of arid C_3_ species to changing climatic conditions. Additionally, iWUE provides a context as to how changing climates, such as the long‐term drying trend in the western USA since 1980 (Zhang et al. 2022) and the more recent megadrought since 2000 (Williams et al. 2020, 2022), may affect the carbon balance within a species over its geographical distribution.

Previous synthesis studies indicated that variations in leaf δ^15^N values reflected both average temperature and soil δ^15^N values (Amundson et al. 1988, 2003; Evans and Ehleringer 1994a, 1994b; Evans 2001, 2007; Craine et al. 2009; Craine, Brookshire, et al. 2015; Craine, Elmore, et al. 2015). These studies highlight the importance of soil microbial processes as influencing both soil δ^15^N transformation and leaf δ^15^N values. Microbial denitrification activities are expected to increase δ^15^N values within nonacidic soil, whereas N_2_ fixation activities are expected to reduce soil δ^15^N values. The Craine, Brookshire, et al. (2015) meta‐analysis of global leaf δ^15^N values concluded that leaf δ^15^N values were positively correlated with growing season temperature and negatively correlated with seasonal precipitation. Thus, a complex climate‐related soil δ^15^N pattern could occur if there were shifts in the balance of these two microbial activities influencing soil δ^15^N content. Most nitrogen loss in desert ecosystems can be attributed to abiotic volatilization under high temperatures, which would favor ^14^N loss as a gas with the resulting N pool ^15^N enriched (McCalley and Sparks 2009; Wang et al. 2014). Kolb and Evans (2003) showed that there was no δ^15^N fractionation by *Encelia* roots during nitrogen uptake from the soil.

Here we have an opportunity to compare data collected from historical and modern observations of leaf δ^13^C and δ^15^N values for *Encelia*. The objectives of this study were to explore historical variations (1972–1980) in leaf δ^13^C and δ^15^N (a) among *Encelia* species, a genus of drought‐deciduous shrubs common to arid habitats in southwestern North America (Shreve and Wiggins 1964; Ehleringer and Clark 1988; Clark 1998) and (b) within *Encelia farinosa*, the most common species in desert ecosystems across Baja California and the Mojave and Sonoran Deserts. These historical *Encelia* observations may be evaluated in terms of current observations (Driscoll et al. 2020; Kannenberg et al. 2021) in response to the ongoing megadrought (Williams et al. 2020, 2022; Cook et al. 2022). We evaluated the extent to which interacting climatic factors across geographic gradients influenced stable isotope ratio relationships.

Here we test three hypotheses: (1) variations in iWUE values among *Encelia* species are positively related to vapor pressure deficit (VPD) and negatively related to precipitation, as has been shown in other taxa; (2) iWUE responses to environmental factors remain indistinguishable among *Encelia* species despite rapid speciation; and (3) δ^15^N values are positively related to habitat temperature.

## 2 Materials and Methods

### 2.1 Sample Collection

**FIGURE 1:**
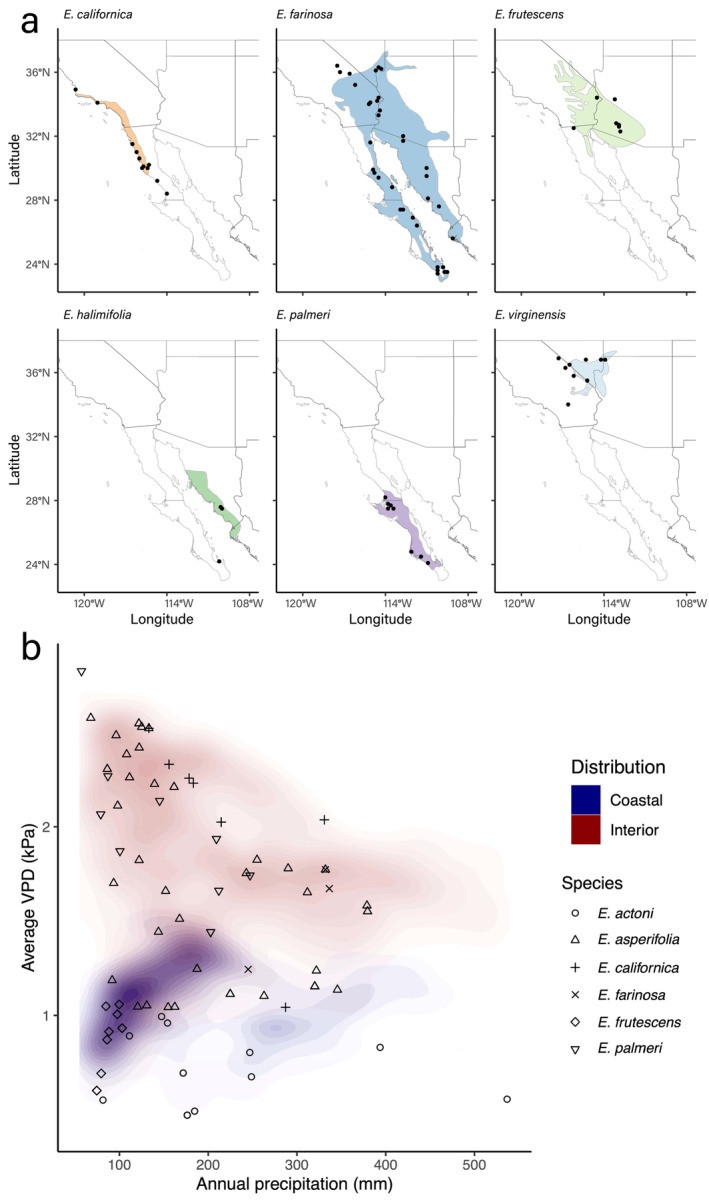
(a) Species distribution maps for the six *Encelia* species included in this study. Range maps were extracted from (Clark 1998). Points represent individual sampling locations. (b) The relationship between annual precipitation and average VPD, with points representing sampling sites and the density contours representing all pixels within the species ranges.

Mature sun leaf samples were randomly collected from one or more *Encelia* plants at 78 sites across their native ranges between 1972 and 1980 (Figure 1). Samples were collected from six *Encelia* species across multiple locations: *E. californica* (*n* = 17), *E. farinosa* (*n* = 48), *E. frutescens* (*n* = 10), *E*. *halimifolia* (*n* = 4), *E. palmeri* (*n* = 17), and *E. virginensis* (*n* = 13). These locations represented the geographical range of *Encelia* across the California coastal chaparral and Mojave and Sonoran Deserts. Leaf samples collected during spring months are hereafter referred to as *spring‐growing* plants and in summer as *summer‐growing* plants.

The distributions of *Encelia* species can be categorized as either occurring along the coast and coastal plains (coastal regions) or within interior regions (Figure 1). The two coastal species exhibit very limited, if any, overlap in distributions. *E. californica* occurs in coastal chaparral vegetation to the north, whereas *E. palmeri* occurs on coastal desert plains to the south. Within the interior landscapes, *E. virginensis* occurs at higher elevations, whereas *E. farinosa* and *E. frutescens* often occur at the intermediate to lower interior elevations. The microdistribution difference is that *E. farinosa* occurs on slopes (drier microhabitat) and *E. frutescens* is confined to washes (wetter microhabitat). The coastal habitats have distinctly lower VPD levels than interior regions, although there is overlap in annual precipitation amounts (Figure 1). The contour lines distinguish these two habitat types.

### 2.2 Sample Processing and Isotopic Analysis

Plant samples were dried in a drying oven at 65°C upon returning from the field and stored in a cool, dark, and dry place until analyses were eventually conducted in 2023. Samples were ground in liquid nitrogen using a mortar and pestle. Samples were then loaded into tin capsules for determinations of δ^13^C, δ^15^N, and %N using a Carlo‐Erba EA‐1110 elemental analyzer coupled to a Finnigan Mat Delta+ IRMS via a continuous flow interface (Conflo III; ThermoFinnigan, Bremen, Germany). Laboratory reference materials were calibrated using international standards USGS40 (δ^13^C = 26.24‰ and δ^15^N = 4.5‰) and USGS41 (δ^13^C = 37.76‰ and δ^15^N = 47.6‰). Results are reported in delta notation relative to VPDB for carbon isotope ratios and atmospheric N_2_ for nitrogen isotope ratios. Long‐term measurements of uncertainty for quality control materials were 0.2‰ for δ^13^C and 0.3‰ for δ^15^N.

### 2.3 iWUE Calculations

Leaf ^13^C values (δ ^13^C_p_) were converted to *c*_*i*_ */c*_*a*_ values according to Equation (1), where δ ^13^C_a_ is the carbon isotope ratio of atmospheric air (Ehleringer and Monson 2025), *a* is the discrimination against ^13^C associated with CO_2_ diffusion (4.4‰), and *b* is the net Rubisco fractionation against ^13^C (27‰).

(1) $$\delta^{13}C_{p}=\delta^{13}C_{a}-a-\left(b-a\right)\frac{c_{i}}{c_{a}}$$

iWUE was then calculated according to Equation (2), where 1.6 is the ratio of the diffusion rates of CO_2_ to H_2_O in air.

(2) $$\text{iWUE}=c_{a}\frac{\left(1-\frac{c_{i}}{c_{a}}\right)}{1.6}$$

*c*_*a*_ and δ^13^C_a_ values vary with time, and values appropriate for the sampling years were used in these calculations.

### 2.4 Climate Data

Monthly climate data for each site were extracted from the TerraClimate database (Abatzoglou et al. 2018), including average precipitation, average minimum temperature, average maximum temperature, and average VPD. To characterize the typical site climate, we calculated the average of each variable over all months between 1964 and 1978 (hereafter referred to as “long‐term average” climate). We also calculated growing season site climate for the sampling year, reflecting the months directly preceding sample collection (hereafter referred to as “seasonal” climate). The summer season was defined as July, August, September, and October; the spring season was defined as November, December, January, February, and March. For example, the “long‐term average” climate values for a site sampled in March of 1972 reflect January 1964 through December 1978, while the “seasonal” climate values reflect November 1971 through March 1972.

### 2.5 Data Analysis

All statistical analyses were conducted in R version 4.4.1 (RStudio Team 2020). First, interspecific variability in iWUE and δ^15^N values among *Encelia* species was assessed using one‐way ANOVA and Tukey's HSD tests. Subsequent linear regression analyses were separated into two categories: all *Encelia* species and *E. farinosa* , which were then analyzed with respect to (a) geography and (b) climate. Geography components included latitude and longitude. Climate analyses were differentiated into three time periods: annual average, spring‐growing season, and summer‐growing season. Within each of the time periods, climate parameters included: (a) minimum, mean, and maximum air temperature, (b) VPD, and (c) precipitation. Samples were analyzed with respect to the growing season in which they were collected.

Isoscapes were constructed using optimized linear regression models, with the full model specification including latitude, longitude, the interaction between latitude and longitude, and long‐term average mean temperature, VPD, and precipitation. Predictors that did not improve model fit, as indicated by the Akaike information criterion (AIC), were iteratively removed from the model. Reduced models were tested for multicollinearity using variance inflation factors (VIF), and insignificant predictors were removed as needed to resolve severe multicollinearity (VIF > 4). For δ^15^N, the optimized model contained longitude, precipitation, mean temperature, and VPD. The optimized models were then used in conjunction with gridded TerraClimate data to predict the expected δ^15^N values in *Encelia* leaves across the species ranges. Because spatial and climatic parameters accounted for little variability in iWUE, isoscapes of iWUE were not created. Spatial analyses were conducted using the R packages “sf,” “stars,” and “terra” (Hijmans 2023; Pebesma and Bivand 2023).

## 3 Results

### 3.1 Geographical Gradients in Climate

*Encelia* species occur in two largely distinct macrohabitats: coastal and interior deserts (Figure 1). To a first approximation, the largest differences between these two macrohabitats are VPD values, with total precipitation amounts often overlapping in paired site coastal‐interior comparisons. To better understand potential constraints imposed by autocorrelations across environmental gradients, we separately regressed climate characteristics. Table 1 provides the results of correlation analyses among the key climate parameters (temperature, precipitation, and VPD) across the California coastal Chaparral and Mojave and Sonoran Desert ecosystems at the sites where *Encelia* samples had been collected. During the spring‐growing season, there was a significant negative correlation between precipitation and VPD (drier locations have higher VPD). During summer‐growing seasons, there were significant positive correlations between temperature versus precipitation and temperature versus VPD (warmer locations are drier).

**TABLE 1:** Correlations among climate parameters.

	Correlation coefficient	*p*
Winter–spring season		
Monthly precipitation vs. mean temperature	0.11	0.45
Monthly precipitation vs. mean VPD	−0.51	< 0.001
Mean temperature vs. mean VPD	0.19	0.19
Summer season		
Monthly precipitation vs. mean temperature	0.50	< 0.001
Monthly precipitation vs. mean VPD	0.21	0.10
Mean temperature vs. mean VPD	0.73	< 0.001

**FIGURE 2:**
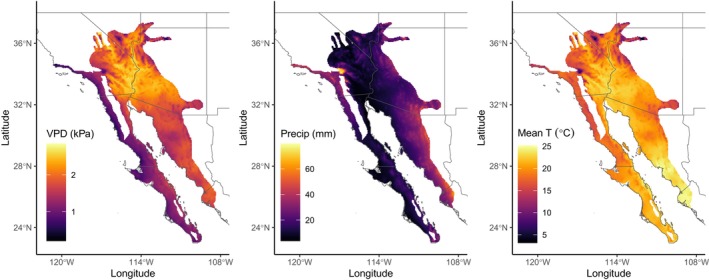
Maps of average (a) VPD (kPa), (b) monthly precipitation, and (c) temperature across the range of the six *Encelia* species.

A multiple regression analysis identified latitude (*L*_*a*_) and longitude (*L*_*o*_) as both significant spatial factors impacting climate parameters (Table 2). The combination of *L*_*a*_ and *L*_*o*_ explained 39% and 58% of the spring‐ and summer‐growing season precipitation patterns, respectively. Precipitation falling during the spring‐growing season (*P*_spr_) is derived from frontal driven storms generated in the Gulf of Alaska, whereas summer‐growing season precipitation (*P*_sum_) arrives as monsoonal moisture emerging from the southern Eastern Pacific Ocean. Together, both precipitation sources contribute to the observed latitudinal gradients in seasonal precipitation, resulting in spring‐growing and summer‐growing seasons. Figure 2 captures the spatial variation in annual precipitation. Here we can see that the driest regions are those in the interior desert, with precipitation increasing to the west as more winter rainfall is received or to the east as more monsoonal precipitation is received.

**TABLE 2:** Climate variations with geography. Latitude (*L*_*a*_) and longitude (*L*_*o*_) were used to define geographic space. Analyses were partitioned into spring and summer seasons. Multiple regressions were calculated with estimates of the contributions of latitude and longitude along with the associated *p* values; before presenting subsequent results. *P*_spr_ and *P*_sum_ are the monthly precipitation during the spring‐growing and summer‐growing seasons, respectively. *T*_spr_ and *T*_sum_ are the mean temperatures during the spring‐growing and summer‐growing seasons, respectively. *T*_mspr_ and *T*_msum_ are the average minimum temperatures during the spring‐growing and summer‐growing seasons, respectively. *T*_xspr_ and *T*_*xsum*_ are the average maximum temperatures during the spring‐growing and summer‐growing seasons, respectively.

Model	Equation	*R* ^2^	*p*
Monthly precipitation during the spring‐growing season (*P*_spr_)	*P*_spr_ = −1615–5.61*L*_*a*_—15.92*L*_*o*_	0.39	< 0.001
Monthly precipitation during the summer‐growing season (*P*_sum_)	*P*_sum_ = 1217 + 3.58*L*_*a*_ + 11.48*L*_*o*_	0.58	< 0.001
Mean VPD during the winter–spring‐growing season (*V*_spr_)	*V*_spr_ = 7.59 + 0.038*L*_*a*_ + 0.069*L*_*o*_	0.28	< 0.001
Mean VPD during the summer‐growing season (*V*_sum_)	*V*_sum_ = 26.99 + 0.265*L*_*a*_ + 0.292*L*_*o*_	0.75	< 0.001
Mean *T*_max_ during the spring‐growing season (*T*_aspr_)	*T*_spr_ = 31.83–0.72*L*_*a*_—0.11*L*_*o*_	0.64	< 0.001
Mean *T*_min_ during the spring‐growing season (*T*_mspr_)	*T*_mspr_ = 39.72–0.55*L*_*a*_ + 0.13*L*_*o*_	0.49	< 0.001
Mean *T*_max_ during the summer‐growing season (*T*_xsum_)	*T*_xsum_ = 120.84 + 0.66*L*_*a*_ + 0.95*L*_*o*_	0.44	< 0.001
Mean *T*_min_ during the summer‐growing season (*T*_msum_)	*T*_msum_ = 216 + 0.63*L*_*a*_ + 1.92*L*_*o*_	0.64	< 0.001

Spatial gradients in VPD are largely explained on the basis of variations in latitude (*L*_*a*_) and longitude (*L*_*o*_) (Table 1). 29% of the geographical variation in VPD (*V*_spr_) during the spring‐growing season was explained by location. Similarly, 75% of the variations in VPD (*V*_sum_) during the summer‐growing season were explained by latitude and longitude, reflecting higher VPD values in northerly latitudes during the summer as well as the pronounced increases in VPD at interior locations. The spatial variations in VPD are presented in Figure 2, where we see that the lowest VPD values are observed along both (a) coastal regions in southern California and northern Baja California and (b) coastal plains in southern Baja California.

Summer growth activities among *Encelia* in southern regions are predominantly driven by monsoonal rains, whereas growth in the northern portions is primarily driven by winter–spring rains (Figure 2). Therefore, for several analyses, we categorized spring‐growing and summer‐growing seasons into northern and southern locations. Here we used 31° N as our spring versus summer rain cutoff latitude, since < 35% of annual rainfall above 31° N is received during summer monsoonal rains (Adams and Comrie 1997; Becker 2024).

### 3.2 Ecophysiological Variations Among All *Encelia* Species

**FIGURE 3:**
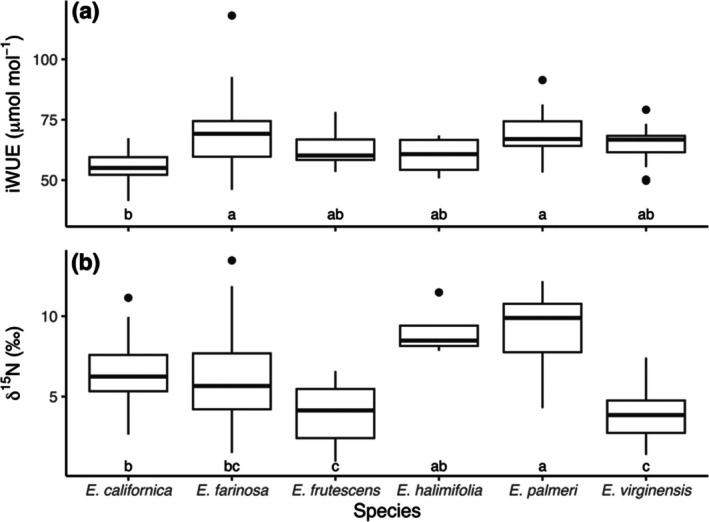
Comparisons of iWUE and leaf δ^15^N values among *Encelia* species. Boxes indicate the first quartile (Q1), median, and third quartile (Q3), and whiskers indicate Q3 and Q1 ± 1.5 × interquartile range, respectively. Letters indicate significant pairwise differences between species based on Tukey's HSD (*α* = 0.05).

We conducted ANOVA analyses among all leaves of all *Encelia* species and identified significant interspecific differences in iWUE (*F* = 5.57, *p* < 0.001) (Figure 3). Across all samples, the mean and standard error of iWUE values were 65.41 ± 1.09 μmol mol^−1^. Notably, *E. californica* occurs in the wettest *Encelia* regions; its iWUE values were significantly different from those of its nearest geographical neighbors, *E. farinosa* (*p* ≤ 0.001) and *E. palmeri* (*p* ≤ 0.001). None of the other *Encelia* species had iWUE values that were significantly different from each other.

We conducted ANOVA analyses among all leaves of all *Encelia* species and identified significant interspecific differences in leaf δ^15^N values (*F* = 10.21, *p* < 0.001) (Figure 3). Across all samples, the mean and standard error of leaf δ^15^N values were 6.30‰ ± 0.27‰. Notably, *E. palmeri* and *E. halimifolia* from the southern Sonoran Desert regions with the greatest fraction of annual precipitation coming as monsoon precipitation had higher δ^15^N values than all other *Encelia* species; though the δ^15^N values of *E. halimifolia* were not statistically different from *E. californica* (*p* = 0.10) or *E. farinosa* (*p* = 0.16).

We next investigated relationships between iWUE and leaf δ^15^N values with climate parameters and with parameters relating to spatial distributions.

#### 3.2.1 iWUE

Year‐round variations in iWUE values among *Encelia* species were significantly related to variations in climate (Table 3; Table S2), yet not significantly related to geography (*p* = 0.147). However, the combination of geography and climate parameters improved model explanatory power (*R* ^2^ = 0.224 vs. 0.112 with climate predictors alone). When the analyses were limited to spring‐growing *Encelia* observations, the model's explanatory power was substantially higher for both (a) climate (*R* ^2^ = 0.430 vs. 0.112) and (b) climate‐geography (*R* ^2^ = 0.494 vs. 0.224) models than when all *Encelia* species were considered (Table 3). None of the model analyses with summer‐growing *Encelia* observations were statistically significant (each had *p* values > 0.22).

**TABLE 3:** Significant spatial relationships occurred among iWUE with climate and geography.

	Parameter	Season	Adjusted *R* ^2^	Model *p*	*N*
iWUE all *Encelia*	Climate	Year‐round	0.112	0.002	107
iWUE all *Encelia*	Climate & Geography	Year‐round	0.224	< 0.001	107
iWUE all *Encelia*	Climate	Spring	0.430	< 0.001	46
iWUE all *Encelia*	Climate & geography	Spring	0.494	< 0.001	45
iWUE *E. farinosa*	Climate	Year‐round	0.187	0.017	39
iWUE *E. farinosa*	Geography	Year‐round	0.181	0.010	39
iWUE *E. farinosa*	Climate & geography	Year‐round	0.341	0.003	37
iWUE *E. farinosa*	Climate	Spring	0.739	< 0.001	18
iWUE *E. farinosa*	Geography	Spring	0.266	0.038	18
iWUE *E. farinosa*	Climate & geography	Spring	0.747	0.001	16
iWUE *E. farinosa*	Climate	Summer	0.247	0.058	20
iWUE *E. farinosa*	Geography	Summer	0.188	0.066	20

Similarly, when model analyses were conducted using only *E. farinosa* observations, we identified that climate and geography were significant drivers of variations in iWUE values with climate and with climate‐geography models during both year‐round and spring‐growing season periods. Almost 74% of the variation among spring‐growing season observations was explained by variations in climate. For summer‐growing season iWUE *E. farinosa* observations, model predictions for both climate‐based and geography‐based parameters were marginally significant predictors of iWUE (*p* = 0.058 and *p* = 0.066, respectively).

#### 3.2.2 δ^15^N

**FIGURE 4:**
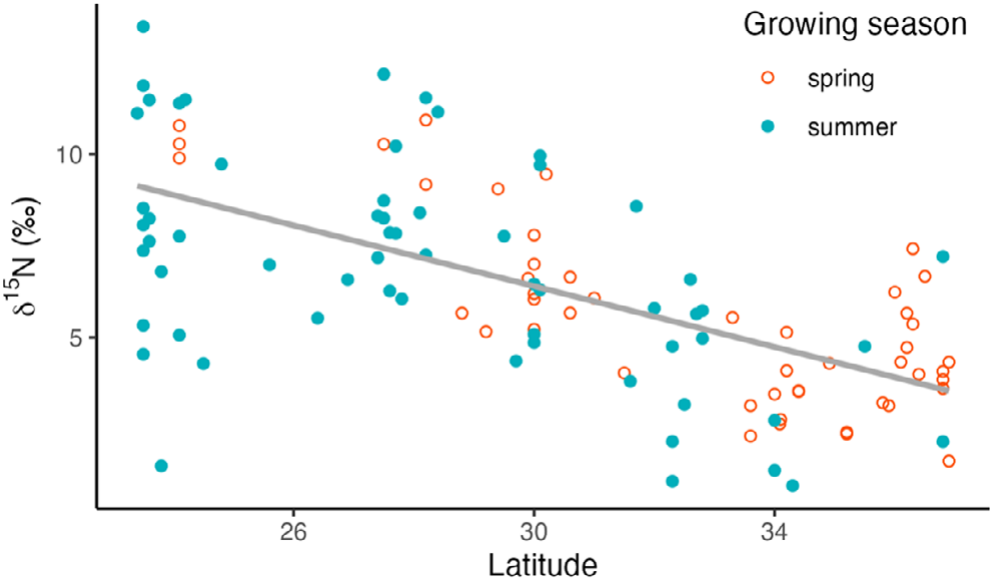
The dependence of leaf δ^15^N values of all *Encelia* leaves on latitude. The different growing‐season observations are shown as (open circles) and summer (closed circles) growing seasons.

Variations in δ^15^N values were significantly related to geography. Leaf δ^15^N values decreased with increasing latitude (*L*_*a*_) (Figure 4), described by δ^15^N = 18.8–0.414*L*_*a*_, *R* ^2^ = 0.394, *p* < 0.0001. In contrast, leaf δ^15^N values increased with longitude, δ^15^N = 0.499*L*_*o*_ + 62.9, *R* ^2^ = 0.183, *p* < 0.0001. In essence, δ^15^N values decreased spatially from the coastal, southwestern portions of *Encelia* distributions (higher δ^15^N) to northern, interior regions (lower δ^15^N values). The model including both climate and geography explained more of the variance (*R* ^2^ = 0.511) than single‐factor regression analyses (Table 4).

**TABLE 4:** Significant spatial relationships occurred among δ^15^N, climate, and geography.

	Parameter	Season	Adjusted *R* ^2^	Model *p*	*N*
δ^15^N all *Encelia*	Climate	Year‐round	0.451	< 0.0001	108
δ^15^N all *Encelia*	Geography	Year‐round	0.445	< 0.0001	108
δ^15^N all *Encelia*	Climate & geography	Year‐round	0.511	< 0.0001	107
δ^15^N all *Encelia*	Climate	Spring	0.623	< 0.0001	48
δ^15^N all *Encelia*	Geography	Spring	0.571	< 0.0001	48
δ^15^N all *Encelia*	Climate & geography	Spring	0.693	< 0.0001	47
δ^15^N all *Encelia*	Climate	Summer	0.363	< 0.0001	61
δ^15^N all *Encelia*	Geography	Summer	0.257	< 0.0001	61
δ^15^N all *Encelia*	Climate & geography	Summer	0.375	< 0.0001	61
δ^15^N *E. farinosa*	Climate	Year‐round	0.580	< 0.0001	38
δ^15^N *E. farinosa*	Geography	Year‐round	0.514	< 0.0001	39
δ^15^N *E. farinosa*	Climate & geography	Year‐round	0.522	< 0.0001	38
δ^15^N *E. farinosa*	Climate	Spring	0.507	0.002	20
δ^15^N *E. farinosa*	Climate & geography	Spring	0.729	0.0003	19

**FIGURE 5:**
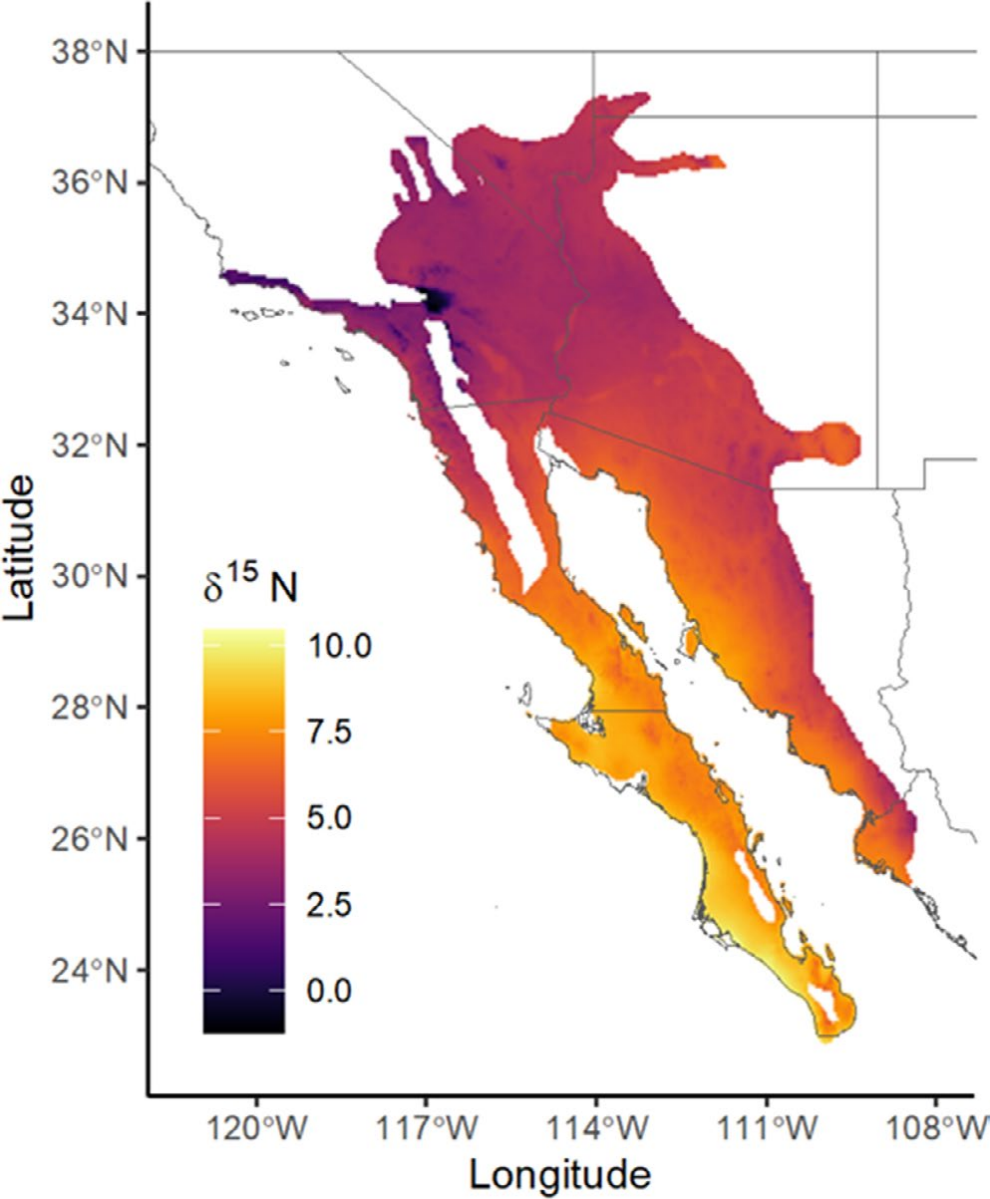
Modeled values of *Encelia* leaf δ^15^N values, based on a multiple linear regression of measured δ^15^N values with longitude and long‐term average precipitation and VPD (*R* ^2^ = 0.543, *p* < 0.0001).

In single factor (Table S2) and multiple linear regression model analyses (Table 4), variations in *Encelia* leaf δ^15^N values were significantly related to climate and geography in each of the combinations of climate, geography, and growing season. That is, when model analyses were conducted using spring‐growing, summer‐growing, and year‐round periods, significant relationships were detected for both climate and geographic parameters. Combining those spatial and climate data parameters influencing *Encelia* δ^15^N values allowed us to produce an isoscape depicting spatial patterns (Figure 5). When all *E. farinosa* observations were analyzed together (year‐round), leaf δ^15^N values were also significantly related to climate and geography (Table 4). Increasing temperatures resulted in higher δ^15^N values. With spring‐growing season samples, modeled δ^15^N values were again significantly related to both climate and geography. However, there were no significant δ^15^N relationships under summer‐growing conditions (all *p* values > 0.281).

**FIGURE 6:**
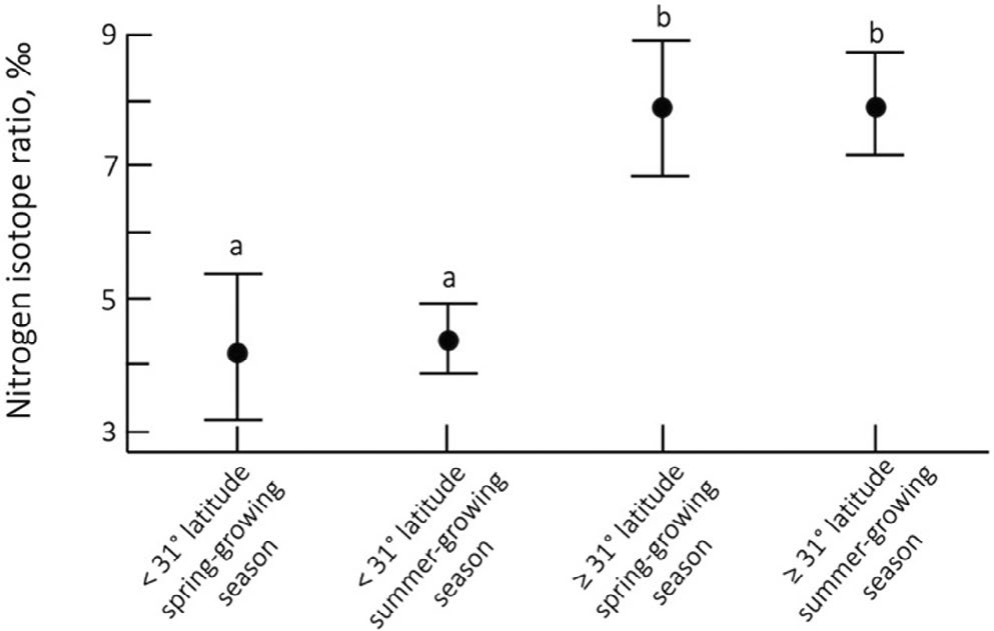
Differences in *Encelia* leaf δ^15^N values (x̄ ± 95% CI) for spring‐growing plants (> 31° N, receiving primary frontal system precipitation) versus summer‐growing plants (< 31° N, receiving primary monsoonal precipitation).

As previously mentioned, precipitation source differences result in more springtime precipitation in northern latitudes and more summer precipitation in southern latitudes. Figure 6 shows that when data were categorized by northern‐ versus southern‐latitudes, *Encelia* δ^15^N values did not statistically differ from one season to the next. Season‐to‐season consistency in leaf δ^15^N values is expected as there is no basis for anticipating large changes in soil microbial activities from year to year. Leaf δ^15^N values from northern latitudes were significantly lower than those from southern latitudes (*F* = 24.19, *p* < 0.001). N_2_‐fixation activities tend to decrease soil δ^15^N values, whereas denitrification activities tend to increase residual soil δ^15^N values. Mean seasonal temperatures (*T*_*s*_) during the spring when northern latitude *Encelia* would on average be expected to be most active averaged 14.5°°C ± 0.99°C (x̄ ± 1.96 × SE). In contrast, southern latitude *Encelia* would on average be expected to be active during the summer when *T*_*s*_ averaged 25.7°C ± 0.77°C.

## 4 Discussion

### 4.1 Why Evaluate *Encelia*?

There are three compelling reasons to explore δ^13^C and δ^15^N variations in *Encelia*. First, *Encelia* is a genus of deciduous‐leaf shrubs common to the arid regions of southwestern North America that has been studied extensively for ecophysiological adaptations to deserts (Ehleringer et al. 1976, 1981; Ehleringer and Mooney 1978; Ehleringer and Cook 1984, 1988, 1990; Smith et al. 1997). These studies have provided morphological (e.g., leaf reflectance and leaf size) evidence consistent with arid land adaptive responses related to leaf temperatures and water relations in a genus that has limited plasticity in thermal adjustments to high temperatures. Its sister genera are *Enceliopsis* (herbaceous perennials) and *Geraea* (annuals), with roughly overlapping distributions across arid ecosystems. Together, the three genera comprise an interesting example of adaptive radiation among closely allied taxa in arid lands, where leaf structure, life form, life history, and canopy architecture differences can provide insights into the tradeoffs associated with adaptive features to contrasting arid conditions. Earlier studies had also established that there was genetic variability (ecotypes) among *E. farinosa* from contrasting climate regions (Schuster et al. 1992, 1994; Sandquist and Ehleringer 1996, 1997, 1998, 2003a, 2003b), but that there is also extensive plasticity in response to interannual variations in temperature, VPD, and water availability (Driscoll et al. 2020; Driscoll, Bitter, and Ehleringer 2021; Driscoll, Kannenberg, and Ehleringer 2021).

Second, Fehlberg and Ranker (2007) provided DNA‐sequence evidence that *Encelia* is not only monophyletic, but they also provided evidence that the genus consists of two major subclades. However, they noted that there is little genetic differentiation among species within each of the subclades. More recently, Singhal et al. (2021) used a nearly complete species‐level phylogeny to show that *Encelia* evolved in the mid‐Pleistocene and then each of the clades rapidly radiated across arid southwestern North America. This rapid evolution within *Encelia* was associated with fast rates of phenotypic radiation, leading to the adaptive variations in leaf size and leaf reflectance seen today. Additionally, all *Encelia* species frequently hybridize in the narrow zones where species overlap (DiVittorio et al. 2020; Singhal et al. 2021).

Third, there is sufficient evidence of plasticity as well as ecotypic differentiation among ecophysiological leaf characteristics within *E. farinosa* (Schuster et al. 1994; Sandquist and Ehleringer 1997, 1998, 2003b) that it may be possible to establish climate‐related patterns incorporating δ^13^C and δ^15^N variations that would allow us to construct isoscapes, which could eventually form the basis for projections as to how *Encelia* species may respond to past conditions, future climate changes, and the current megadrought still underway. In many ways, we have approached this study with an implicit concept that there are few, if any, barriers to gene flow within *Encelia*, which has been supported by previous studies (Fehlberg and Ranker 2009; Fehlberg and Fehlberg 2017; Singhal et al. 2021).

### 4.2 iWUE and Climate

Hypotheses 1 and 2 describe expected ecophysiological responses to water‐related climate parameters: (1) *variations in iWUE values among Encelia species are positively related to vapor pressure deficit (VPD), and negatively related to precipitation*; and (2) *while Encelia species have evolved into distinct species, iWUE responses to environmental factors remain indistinguishable despite rapid speciation*. Figure 1 shows that, across spring‐ and summer‐growing regions, the distributions of *Encelia* species can be ordinated along precipitation (water availability) and VPD (evaporative demand basis) axes in order to evaluate these hypotheses. Here, Hypothesis 1 predicted that iWUE in *Encelia* would increase with decreasing precipitation. The climate analyses in Table 3 and Table S2 support this hypothesis, with iWUE increasing among all *Encelia* as growing season precipitation decreased. Since our sample sizes were only sufficient to evaluate species‐level iWUE trends in *E. farinosa* , it is noteworthy that we also found support for this hypothesis with a negative relationship between iWUE and growing season precipitation for plants in both growing seasons (*p* = 0.010) and spring‐growing plants (*p* = 0.004). However, there were no statistically significant relationships between iWUE and summer growing season precipitation among either all *Encelia* or *E. farinosa* leaves. With respect to the second part of Hypothesis 1, the data in Tables 3 and Table S2 revealed that iWUE values were positively related to long‐term average VPD among all *Encelia* in both growing seasons (*p* = 0.039) and for spring‐growing season *Encelia* (*p* = 0.028). These trends imply stomatal conductance reductions with increasing VPD values. As there were also significant trends with respect to iWUE and temperature, multiple linear regressions incorporating precipitation, VPD, and temperature explained a greater fraction of the variance than did precipitation or VPD alone (Table 3; Table S2). Thus, our predictions for Hypothesis 1 were supported. Similar patterns have been noted by Driscoll et al. (2020), Driscoll, Kannenberg, and Ehleringer (2021), and Kannenberg et al. (2021).

Arid portions of southwestern North America have become increasingly drier in the decades since the 1972–1980 period during which the samples in this study were collected (Williams et al. 2020, 2022; Cook et al. 2022, 2025; Seager et al. 2023). Four decades later between 2010 and 2020, the average *E. farinosa and E. frutescens* iWUE values increased from 89 to 105 μmol CO_2_ mol^−1^ with individual observations now exceeding 120 μmol CO_2_ mol^−1^ H_2_O (Kannenberg et al. 2021). These recent iWUE observations far exceed the average *E. farinosa and E. frutescens* iWUE values of 69 and 63, respectively, in the 1972–1980 observation period of this study when average VPD values were lower (Figure 2). Yet iWUE versus VPD patterns today (Driscoll et al. 2020; Kannenberg et al. 2021; Ehleringer and Driscoll 2022) extrapolate back to the same average, paired *E. farinosa* iWUE‐VPD values measured four decades earlier. This likely implies that there were no appreciable genetic changes in the ecophysiological relationships over the past five decades, but more likely changes in expressed plasticity. While it is unknown just how high iWUE values in viable *E. farinosa* leaves can rise, it is noteworthy that the current multi‐decade megadrought is still underway (Cook et al. 2025). Moreover, the combination of exceptionally high VPD and low precipitation values in the Mojave Desert between 2019 and 2022 has resulted in extensive *E. farinosa* mortality (Ehleringer, in preparation). Lastly, the Kannenberg et al. (2021) study noted that the impacts of rising VPD during the current megadrought exceeded the impacts of decreasing precipitation levels, suggesting that future regional VPD levels may continue to have the impact of decreasing iWUE values and likely also negatively impact primary productivity and plant survival. The recent analyses by Fang et al. (2022) of global increases in VPD values over the last four decades portend possible greater long‐term increases in *Encelia* iWUE, even if drought under the current megadrought is relieved. As there are likely limits to which C_3_ plants tolerate high iWUE values (Farquhar et al. 1989; Cernusak et al. 2013) coincident with likely decreases in primary productivity, subsequent studies could experimentally explore upper limits to iWUE values within this common shrub species. Such studies could provide predictions of restricted *Encelia* distributions within the most arid portions of the Mojave and Sonoran Deserts.

Given the abundances and widespread distribution of *E. farinosa* , long‐term population dynamics under climate variations and longevity have been the focus of frequent studies over the last century (Shreve and Hinckley 1937; Goldberg and Turner 1986; Bowers 2005; Ehleringer and Sandquist 2018; Driscoll et al. 2020; Driscoll, Bitter, and Ehleringer 2021; Kannenberg et al. 2021; Ehleringer and Driscoll 2022). Our evidence is of long‐term reductions in population size since 1980, with drought favoring individuals with higher iWUE values than lower iWUE values (Ehleringer 1993; Sandquist and Ehleringer 1997, 1998, 2003a, 2003b; Ehleringer and Driscoll 2022).

### 4.3 δ^15^N and Climate

Syntheses of soil nitrogen cycling conclude that soil δ^15^N values reflect, in part, a balance between two processes having opposing effects on δ^15^N values: (a) biological N_2_ fixation activities that lower soil δ^15^N values and increase soil N content versus (b) microbial denitrification activities and abiotic loss pathways that enrich soil δ^15^N values and decrease soil N content (Högberg 1997; Robinson 2001; McCalley and Sparks 2009). Additionally, previous studies have shown that leaf δ^15^N values were a good proxy for soil δ^15^N (Evans and Ehleringer 1993; Craine, Brookshire, et al. 2015), though additional fractionations can occur during N uptake, storage, reallocation, and losses (Evans 2001; Kolb and Evans 2003). Despite these complexities, we approach our interpretation of *Encelia* leaf δ^15^N observations with the implicit assumption that leaf δ^15^N data reflect relative balances among those microbial processes increasing versus decreasing soil N contents (i.e., N_2_‐fixation versus denitrification).

Hypothesis 3 stated *δ* ^*15*^ *N values are positively related to habitat temperature*. Our observations confirm this hypothesis with *Encelia* leaf δ^15^N values positively related to mean seasonal temperatures among all *Encelia* in both spring‐ (*p* = 0.0007) and summer‐growing seasons (*p* = 0.009). In addition, our analyses revealed an unexpected pattern: *Encelia* and spring‐growing season *E. farinosa* leaf δ^15^N values were also dependent on VPD (both *p* < 0.0001). Partitioning these analyses into spring‐ versus summer‐growing regions clarified these patterns, illustrating that leaf δ^15^N values of plants from these contrasting growing seasons exhibited significantly different values.

Precipitation in the Mojave and Sonoran Deserts is determined by one of two oceanic moisture sources: winter frontal systems originating from the Northern Pacific Ocean and summer‐monsoon systems originating from the central Eastern Pacific Ocean (Sheppard et al. 2002). A latitude of 31° N represents a geographical transition from one primary moisture source to the other (Adams and Comrie 1997; Becker 2024). With respect to these boundaries, all *Encelia* from spring‐growing regions (31°–37° N) exhibited significantly lower δ^15^N values than summer‐growing *Encelia* (23.4°–30.9° N). The contrasting δ^15^N difference between growing regions of 3 + ‰ implied a significant difference in the balance of soil microbial N cycling activities. Soils in cooler northern regions had lower leaf δ^15^N values, indicating a shift toward greater soil N_2_ fixation than in the warmer soils in southern latitudes. The consistency in observed leaf δ^15^N values when sampled in either spring or summer periods provided strong support for the notion that soil δ^15^N values did not appreciably change throughout the year.

While southern regions of the Sonoran Desert have higher temperatures on an annual basis, these regions are not as warm during the summer as northern latitudes. Here during the summer in the southern regions, monsoonal precipitation allows for great energy dissipation of absorbed energy by the soil through latent heat transfer. In contrast, in the northern regions, that equivalent heat load must be dissipated through increased temperatures and sensible heat loss since summers are dry periods. Cooler spring temperatures in northern latitudes, when soil N_2_ fixation likely occurs following winter–spring rains, result in soils during the hottest summer periods when both plants and soil microbes are more likely to be inactive. In contrast, plants from southern latitudes occur in soils exposed to year‐round warm temperatures. This tendency for northern latitudes to have lower leaf δ^15^N values because of spring activities is reflected in the δ^15^N isoscape (Figure 5).

As we search to provide a basis for latitudinal δ^15^N variations, we note that none of the *Encelia* species are known to have N_2_‐fixing nodules or established relationships with free‐living N_2_‐fixing bacteria. As is common in deserts, nutrient levels are low and mature shrubs tend to serve as relative “islands of fertility” with low nutrient concentrations between plants (Noy‐Meir 1985; Schlesinger et al. 1996; Driscoll, Kannenberg, and Ehleringer 2021). It is likely that free‐living *Rhizobium* utilize litter that has accumulated below shrubs as a carbon source since *E. farinosa* leaf δ^15^N values (and the soils below shrubs) are also lower in these islands of fertility (Driscoll, Kannenberg, and Ehleringer 2021). However, these observations do not provide an explanation for why leaf δ^15^N values are higher in southern latitudes, except that denitrification rates must be greater there.

### 4.4 Spatial Patterns

This study has shown that there are spatial patterns in iWUE values (calculated from δ^13^C) and δ^15^N values among *Encelia*. Yet no species‐specific spatial patterns emerged as were predicted by Hypothesis 3. All *Encelia* species followed the same climatic relationships. Spatially, iWUE increased with latitude, whereas δ^15^N values decreased with latitude. The influences of climate drivers could be established for variations in both iWUE and δ^15^N values. In terms of ecophysiological responses, the well‐known influences of precipitation (soil drought) and VPD (atmospheric drought) on stomatal activities explained conditions under which iWUE increased. While δ^15^N values decreased with latitude, the mechanistic driver was not established, other than to indicate that variations in *Encelia* δ^15^N reflected likely shifts in soil N_2_‐fixation versus denitrification activities that were temperature dependent.

## Conflicts of Interest

The authors declare no conflicts of interest.

## Supporting information


**Table S1:** This table provides all individual species, location, δ^13^C and δ^15^N observations, and extracted climate data used in these analyses. All single‐parameter climate and geographical regressions for iWUE and leaf δ^15^N values are provided in Table S2.

**Table S2:** Values from single factor regression of climate and geographical parameters influencing iWUE and nitrogen isotope ratios.

## Data Availability

All data used in these analyses are provided in Supporting Information.
