# Supplementary material for: Climate Gradients Underlie Geographical Variations in iWUE and δ 15N Values of Encelia
Source: Plant Environ Interact. 2025 Aug 28;6(4):e70080. doi: 10.1002/pei3.70080 (PMC12391729; doi:10.1002/pei3.70080)
Supplement: Supplementary file 1 — Table S1: This table provides all individual species, location, δ13C and δ15N observations, and extracted climate data used in these analyses. All single‐parameter climate and geographical regressions for iWUE and leaf δ15N values are provided in Table S2. [file PEI3-6-e70080-s001.pdf]

genus	species	latitude, °N	longitude, °E	altitude, m	date	year	season	measured d13C, ‰	extracted atmospheric CO2, ppm	extracted d13Cair, ‰	calculated ci/ca	calculated iWUE µmol mol-1	measured d15N, ‰	measured leaf N, %	extracted average annual precipitation, mm	extracted seasonal precipitation, mm	extracted seasonal total precipitation, mm	extracted average minimum temperature, °C	extracted average maximum temperature, °C	extracted seasonal minimum temperature, °C	extracted seasonal maximum temperature, °C	extracted seasonal average temperature, °C	extracted average VPD kPa Pa-1	extracted seasonal VPD, kPa Pa-1
Encelia	californica	28.4	-114	30	10/16/74	1974	summer	-28.2	330.3	-7.22	0.733	55.0	11.1	3.3	81	2	10	8.77	25.79	11.60	29.50	20.55	0.55	0.37
Encelia	californica	29.2	-114.7	30	2/26/78	1978	spring	-28.7	335.5	-7.34	0.752	52.0	5.2	3.0	111	43	172	10.56	27.69	10.76	25.18	17.97	0.89	0.97
Encelia	californica	30.2	-115.3	350	2/27/78	1978	spring	-28.4	335.5	-7.34	0.738	55.0	9.4	4.1	148	50	199	8.76	26.12	7.33	21.99	14.66	0.99	0.77
Encelia	californica	30	-115.4	25	10/16/74	1974	summer	-27.6	330.3	-7.22	0.709	60.1	6.5	2.7	154	11	43	9.04	26.37	13.77	31.87	22.82	0.96	1.21
Encelia	californica	30.1	-115.7	15	10/16/74	1974	summer	-29.7	330.3	-7.22	0.800	41.4	9.9	4.2	172	8	32	8.58	25.13	12.41	29.29	20.85	0.69	0.73
Encelia	californica	30.1	-115.7	60	10/29/75	1975	summer	-27.1	331.2	-7.25	0.684	65.4	9.7	3.2	172	1	4	8.58	25.13	12.25	28.92	20.58	0.69	0.70
Encelia	californica	30.1	-115.7	15	10/16/74	1974	summer	-28.0	330.3	-7.22	0.726	56.5	6.3	4.1	172	8	32	8.58	25.13	12.41	29.29	20.85	0.69	0.73
Encelia	californica	30	-115.8	15	2/26/78	1978	spring	-29.1	335.5	-7.34	0.769	48.5	6.2	4.5	176	79	318	7.65	23.86	8.40	22.71	15.55	0.47	0.68
Encelia	californica	30	-115.8	15	2/26/78	1978	spring	-28.5	335.5	-7.34	0.743	53.9	7.0	3.6	176	79	318	7.65	23.86	8.40	22.71	15.55	0.47	0.68
Encelia	californica	30	-115.8	15	2/26/78	1978	spring	-27.8	335.5	-7.34	0.712	60.4	6.0	3.4	176	79	318	7.65	23.86	8.40	22.71	15.55	0.47	0.68
Encelia	californica	30	-115.8	244	2/27/78	1978	spring	-27.1	335.5	-7.34	0.679	67.3	5.2	3.3	176	79	318	7.65	23.86	8.40	22.71	15.55	0.47	0.68
Encelia	californica	30.6	-116	61	2/26/78	1978	spring	-28.3	335.5	-7.34	0.732	56.2	5.7	3.8	185	87	346	7.55	23.44	7.23	22.08	14.65	0.49	0.61
Encelia	californica	30.6	-116	61	2/26/78	1978	spring	-29.2	335.5	-7.34	0.771	47.9	6.6	3.7	185	87	346	7.55	23.44	7.23	22.08	14.65	0.49	0.61
Encelia	californica	27.4	-112.9	215	10/17/74	1974	summer	-28.1	330.3	-7.22	0.728	56.2	7.2	1.3	187	22	89	11.73	28.71	16.86	33.97	25.41	1.25	1.48
Encelia	californica	31.5	-116.5	61	2/26/78	1978	spring	-28.6	335.5	-7.34	0.748	52.9	4.0	4.2	247	103	412	9.82	23.40	8.55	21.34	14.94	0.80	0.73
Encelia	californica	31	-116.2	25	2/26/78	1978	spring	-28.9	335.5	-7.34	0.757	50.9	6.1	4.9	249	111	444	8.43	24.04	7.28	22.39	14.84	0.67	0.70
Encelia	californica	34.1	-119.04	30	2/29/76	1976	spring	-27.7	332.2	-7.28	0.707	60.8	2.6	5.3	394	26	102	9.97	21.70	6.17	20.40	13.29	0.83	0.83
Encelia	californica	34.9	-120.6	76	6/23/78	1978	spring	-28.1	335.5	-7.34	0.725	57.6	4.3	3.1	537	172	687	8.14	18.89	8.77	18.76	13.77	0.56	0.57
Encelia	farinosa	35.9	-116.6	444	3/16/78	1978	spring	-27.5	335.5	-7.34	0.698	63.3	3.1	2.7	68	19	74	14.09	30.29	7.55	22.09	14.82	2.58	1.14
Encelia	farinosa	35.2	-116.2	366	3/18/78	1978	spring	-26.9	335.5	-7.34	0.670	69.3	2.4	1.6	86	20	81	12.76	28.37	6.77	20.08	13.43	2.31	1.05
Encelia	farinosa	35.2	-116.2	366	3/18/78	1978	spring	-26.5	335.5	-7.34	0.653	72.8	2.4	1.3	86	20	81	12.76	28.37	6.77	20.08	13.43	2.31	1.05
Encelia	farinosa	28.8	-113.5	61	3/1/78	1978	spring	-26.7	335.5	-7.34	0.662	70.8	5.7	1.4	92	18	70	12.02	29.12	9.44	24.17	16.80	1.19	0.86
Encelia	farinosa	31.6	-115.1	15	10/26/74	1974	summer	-26.6	330.3	-7.22	0.665	69.2	3.8	1.9	94	8	33	12.27	29.50	18.48	35.67	27.07	1.70	2.28
Encelia	farinosa	34.1	-115.1	60	4/20/75	1975	spring	-24.4	331.2	-7.25	0.563	90.5	2.8	2.1	96	4	18	15.39	29.52	6.40	19.88	13.14	2.49	1.11
Encelia	farinosa	36	-117.3	705	4/4/75	1975	spring	-24.1	331.2	-7.25	0.552	92.7	6.2	1.9	98	8	31	11.09	27.35	2.59	17.75	10.17	2.11	0.88
Encelia	farinosa	34	-115.2	215	4/20/75	1975	spring	-25.0	331.2	-7.25	0.592	84.4	3.5	2.8	108	5	18	15.08	28.91	6.52	19.70	13.11	2.38	1.12
Encelia	farinosa	36.1	-114.7	549	2/9/78	1978	spring	-27.7	335.5	-7.34	0.707	61.4	4.3	2.0	111	26	104	13.65	27.59	8.39	20.09	14.24	2.26	1.11
Encelia	farinosa	29.7	-114.8	675	10/29/75	1975	summer	-25.7	331.2	-7.25	0.620	78.6	4.4	2.1	121	2	6	9.63	26.98	15.10	32.43	23.76	1.05	1.33
Encelia	farinosa	34.2	-114.6	213	3/19/78	1978	spring	-28.2	335.5	-7.34	0.730	56.7	4.1	3.2	122	28	112	15.24	29.94	9.20	21.79	15.50	2.55	1.25
Encelia	farinosa	34.2	-114.6	213	3/19/78	1978	spring	-27.1	335.5	-7.34	0.680	67.0	5.1	3.6	122	28	112	15.24	29.94	9.20	21.79	15.50	2.55	1.25
Encelia	farinosa	36.4	-117.5	830	4/3/75	1975	spring	-21.4	331.2	-7.25	0.429	118.1	4.0	2.0	122	10	40	8.93	24.91	1.14	15.64	8.39	1.82	0.79
Encelia	farinosa	33.3	-114.5	183	3/19/78	1978	spring	-26.4	335.5	-7.34	0.647	74.1	5.5	1.9	122	23	91	14.61	30.09	9.14	22.40	15.77	2.42	1.26
Encelia	farinosa	33.6	-114.4	NA	4/20/75	1975	spring	-25.4	331.2	-7.25	0.607	81.4	2.3	2.6	125	5	20	14.69	30.62	5.94	21.36	13.65	2.53	1.19
Encelia	farinosa	33.6	-114.4	245	4/20/75	1975	spring	-26.4	331.2	-7.25	0.651	72.3	3.1	3.6	125	5	20	14.69	30.62	5.94	21.36	13.65	2.53	1.19
Encelia	farinosa	29.9	-114.9	610	2/27/78	1978	spring	-28.2	335.5	-7.34	0.729	56.8	6.6	4.0	131	37	149	8.94	26.25	7.34	21.43	14.39	1.05	0.77
Encelia	farinosa	34.4	-114.5	610	3/19/78	1978	spring	-27.8	335.5	-7.34	0.711	60.6	3.5	2.5	133	30	119	15.12	29.46	8.92	21.18	15.05	2.52	1.21
Encelia	farinosa	36.3	-114.5	488	2/9/78	1978	spring	-26.6	335.5	-7.34	0.656	72.1	5.4	4.5	140	31	123	13.00	26.49	6.68	17.66	12.17	2.23	0.93
Encelia	farinosa	27.4	-112.7	150	10/17/74	1974	summer	-26.7	330.3	-7.22	0.666	68.9	8.3	2.2	144	17	69	13.48	30.46	18.85	35.64	27.25	1.44	1.72
Encelia	farinosa	26.9	-112	15	10/17/74	1974	summer	-27.7	330.3	-7.22	0.712	59.4	6.6	2.3	152	20	78	16.43	31.27	23.05	36.07	29.56	1.66	2.01
Encelia	farinosa	29.4	-114.5	NA	3/1/78	1978	spring	-25.6	335.5	-7.34	0.612	81.3	9.0	2.1	155	44	175	9.42	26.20	7.97	21.60	14.78	1.04	0.81
Encelia	farinosa	36.2	-114.3	640	2/7/78	1978	spring	-28.3	335.5	-7.34	0.734	55.7	4.7	2.4	162	36	143	12.71	26.41	6.59	17.52	12.06	2.21	0.94
Encelia	farinosa	36.2	-114.3	442	2/7/78	1978	spring	-28.0	335.5	-7.34	0.721	58.6	5.7	3.8	162	36	143	12.71	26.41	6.59	17.52	12.06	2.21	0.94
Encelia	farinosa	23.4	-110.2	30	10/20/74	1974	summer	-27.9	330.3	-7.22	0.722	57.4	11.1	1.5	162	22	88	15.77	28.46	21.64	32.23	26.93	1.05	1.27
Encelia	farinosa	26.4	-111.7	60	10/18/74	1974	summer	-28.0	330.3	-7.22	0.724	56.9	5.5	1.7	168	21	84	15.35	30.19	21.35	34.88	28.12	1.51	1.81
Encelia	farinosa	30	-115.8	15	2/27/78	1978	spring	-28.6	335.5	-7.34	0.747	53.0	7.8	3.3	176	79	318	7.65	23.86	8.40	22.71	15.55	0.47	0.68
Encelia	farinosa	23.6	-110.2	NA	11/5/75	1975	summer	-27.3	331.2	-7.25	0.691	64.0	11.5	2.4	225	32	127	15.01	28.44	20.18	31.87	26.03	1.11	1.29
Encelia	farinosa	23.6	-110.2	185	10/20/74	1974	summer	-27.7	330.3	-7.22	0.712	59.4	8.2	2.1	225	34	135	15.01	28.44	20.63	31.84	26.23	1.11	1.28
Encelia	farinosa	23.6	-110.2	250	10/20/74	1974	summer	-29.2	330.3	-7.22	0.777	46.0	7.6	1.8	225	34	135	15.01	28.44	20.63	31.84	26.23	1.11	1.28
Encelia	farinosa	31.7	-112.7	460	10/25/74	1974	summer	-27.9	330.3	-7.22	0.718	58.2	8.6	2.1	243	46	184	11.57	28.57	19.45	35.18	27.31	1.75	2.35
Encelia	farinosa	32	-112.7	705	11/6/77	1977	summer	-25.7	334.1	-7.31	0.621	79.2	5.8	4.6	255	39	157	12.04	28.09	20.93	35.59	28.26	1.82	2.58
Encelia	farinosa	23.8	-110.2	275	10/20/74	1974	summer	-26.0	330.3	-7.22	0.634	75.6	6.8	2.8	263	40	160	14.30	28.07	19.87	31.47	25.67	1.10	1.26
Encelia	farinosa	28.1	-110.9	61	10/24/74	1974	summer	-27.7	330.3	-7.22	0.710	59.9	8.4	1.5	290	58	231	18.39	30.60	24.41	34.57	29.49	1.78	1.90
Encelia	farinosa	25.6	-109.1	1.5	10/23/74	1974	summer	-29.1	330.3	-7.22	0.773	46.9	7.0	2.6	312	60	241	15.98	32.25	22.34	34.95	28.65	1.65	1.79
Encelia	farinosa	23.5	-109.5	15	10/21/74	1974	summer	-26.0	330.3	-7.22	0.638	74.8	8.1	1.6	320	58	232	15.29	29.17	21.60	32.65	27.13	1.15	1.37
Encelia	farinosa	23.5	-109.5	60	11/1/75	1975	summer	-26.4	331.2	-7.25	0.651	72.2	4.5	2.8	320	41	165	15.29	29.17	21.17	32.53	26.85	1.15	1.36
Encelia	farinosa	23.5	-109.5	60	11/2/75	1975	summer	-25.8	331.2	-7.25	0.628	77.0	13.5	3.6	320	41	165	15.29	29.17	21.17	32.53	26.85	1.15	1.36
Encelia	farinosa	23.5	-109.5	60	11/2/75	1975	summer	-26.6	331.2	-7.25	0.662	70.0	11.9	2.6	320	41	165	15.29	29.17	21.17	32.53	26.85	1.15	1.36
Encelia	farinosa	23.5	-109.5	25	11/5/75	1975	summer	-26.0	331.2	-7.25	0.637	75.2	8.5	3.2	320	41	165	15.29	29.17	21.17	32.53	26.85	1.15	1.36
Encelia	farinosa	23.5	-109.5	60	10/21/74	1974	summer	-26.3	330.3	-7.22	0.651	72.0	7.4	1.5	320	58	232	15.29	29.17	21.60	32.65	27.13	1.15	1.37
Encelia	farinosa	23.5	-109.7	60	10/21/74	1974	summer	-26.8	330.3	-7.22	0.673	67.5	5.3	1.2	322	61	246	15.71	29.70	22.09	33.33	27.71	1.24	1.50
Encelia	farinosa	27.6	-110.1	NA	10/23/74	1974	summer	-27.3	330.3	-7.22	0.693	63.4	6.3	2.1	332	74	294	16.68	31.75	23.30	35.01	29.15	1.77	1.85
Encelia	farinosa	23.8	-109.8	490	10/22/74	1974	summer	-27.5	330.3	-7.22	0.701	61.7	1.5	2.0	346	61	243	13.75	28.31	19.62	31.43	25.52	1.14	1.27
Encelia	farinosa	29.5	-111	520	10/24/74	1974	summer	-26.8	330.3	-7.22	0.670	68.0	7.8	2.6	379	69	274	12.71	28.75	19.44	32.92	26.18	1.58	1.72
Encelia	farinosa	30	-111	NA	11/8/75	1975	summer	-26.4	331.2	-7.25	0.655	71.5	5.1	2.5	380	62	248	11.42	28.24	18.16	33.53	25.85	1.55	1.89
Encelia	farinosa	30	-111	NA	11/8/75	1975	summer	-26.2	331.2	-7.25	0.644	73.6	4.9	3.1	380	62	248	11.42	28.24	18.16	33.53	25.85	1.55	1.89
Encelia	frutescens	34.4	-114.5	610	3/19/78	1978	spring	-28.0	335.5	-7.34	0.719	58.9	3.5	3.8	133	30	119	15.12	29.46	8.92	21.18	15.05	2.52	1.21

Encelia	frutescens	32.8	-113.1	615	9/9/76	1976	summer	-27.4	332.2	-7.28	0.696	63.2	5.7	2.4	156	25	102	14.76	30.30	22.15	36.95	29.55	2.33	3.16
Encelia	frutescens	32.8	-113.1	615	9/9/76	1976	summer	-27.7	332.2	-7.28	0.711	60.0	5.0	3.1	156	25	102	14.76	30.30	22.15	36.95	29.55	2.33	3.16
Encelia	frutescens	32.7	-112.9	275	11/7/77	1977	summer	-28.4	334.1	-7.31	0.740	54.3	5.6	2.7	178	22	88	14.52	29.80	24.07	38.13	31.10	2.26	3.35
Encelia	frutescens	32.6	-112.9	305	11/7/77	1977	summer	-28.0	334.1	-7.31	0.721	58.2	6.6	3.3	183	24	94	14.66	29.61	24.10	37.93	31.01	2.23	3.30
Encelia	frutescens	32.3	-112.8	488	11/7/77	1977	summer	-27.8	334.1	-7.31	0.711	60.3	4.8	2.1	215	29	115	14.28	28.43	23.29	36.41	29.85	2.02	2.94
Encelia	frutescens	32.3	-112.8	NA	11/7/77	1977	summer	-26.9	334.1	-7.31	0.674	68.0	2.2	0.7	215	29	115	14.28	28.43	23.29	36.41	29.85	2.02	2.94
Encelia	frutescens	32.3	-112.8	NA	11/7/77	1977	summer	-25.8	334.1	-7.31	0.626	78.2	1.1	0.6	215	29	115	14.28	28.43	23.29	36.41	29.85	2.02	2.94
Encelia	frutescens	32.5	-116.2	914	9/9/76	1976	summer	-28.5	332.2	-7.28	0.743	53.4	3.2	2.8	287	35	138	6.24	20.51	12.13	26.48	19.30	1.04	1.48
Encelia	frutescens	34.3	-113.2	640	11/5/77	1977	summer	-26.3	334.1	-7.31	0.645	74.1	1.0	2.6	331	31	124	9.73	27.60	19.02	36.03	27.52	2.04	3.04
Encelia	halimifolia	24.2	-110.2	15	11/1/75	1975	summer	-27.0	331.2	-7.25	0.681	66.0	11.5	2.6	245	33	130	15.25	28.86	20.66	32.92	26.79	1.24	1.52
Encelia	halimifolia	27.6	-110.1	75	10/23/74	1974	summer	-26.7	330.3	-7.22	0.668	68.5	7.9	2.8	332	74	294	16.68	31.75	23.30	35.01	29.15	1.77	1.85
Encelia	halimifolia	27.5	-110	75	10/23/74	1974	summer	-28.7	330.3	-7.22	0.754	50.8	8.2	3.0	336	75	299	15.91	31.31	23.10	35.09	29.10	1.67	1.85
Encelia	halimifolia	27.5	-110	75	10/23/74	1974	summer	-28.2	330.3	-7.22	0.731	55.4	8.7	2.5	336	75	299	15.91	31.31	23.10	35.09	29.10	1.67	1.85
Encelia	palmeri	28.2	-114	30	10/30/75	1975	summer	-27.8	331.2	-7.25	0.713	59.4	7.2	1.7	75	0	2	9.22	26.15	13.07	29.83	21.45	0.60	0.50
Encelia	palmeri	28.2	-114	30	2/28/78	1978	spring	-27.4	335.5	-7.34	0.692	64.6	9.2	1.6	75	20	78	9.22	26.15	8.71	23.25	15.98	0.60	0.65
Encelia	palmeri	28.2	-114	30	10/16/74	1974	summer	-26.1	330.3	-7.22	0.640	74.4	11.5	2.7	75	2	7	9.22	26.15	12.34	30.14	21.24	0.60	0.48
Encelia	palmeri	28.2	-114	30	2/28/78	1978	spring	-25.6	335.5	-7.34	0.612	81.3	10.9	2.2	75	20	78	9.22	26.15	8.71	23.25	15.98	0.60	0.65
Encelia	palmeri	24.8	-112.1	1.5	10/18/74	1974	summer	-26.4	330.3	-7.22	0.654	71.3	9.7	1.7	80	6	24	14.05	25.41	18.89	29.36	24.13	0.69	0.79
Encelia	palmeri	24.5	-111.4	107	10/19/74	1974	summer	-26.4	330.3	-7.22	0.654	71.4	4.3	2.3	85	9	37	15.89	27.61	20.96	31.41	26.19	1.05	1.23
Encelia	palmeri	27.5	-113.8	15	2/28/78	1978	spring	-27.4	335.5	-7.34	0.694	64.2	10.3	3.1	86	18	73	11.12	27.98	9.92	24.85	17.39	0.87	0.81
Encelia	palmeri	27.8	-113.8	46	10/17/74	1974	summer	-27.1	330.3	-7.22	0.687	64.7	6.1	2.8	88	4	17	11.20	28.17	15.13	32.80	23.97	0.91	0.97
Encelia	palmeri	27.7	-113.6	76	10/30/75	1975	summer	-26.4	331.2	-7.25	0.653	71.8	7.8	2.5	98	2	6	11.56	28.71	16.35	33.28	24.82	1.01	1.12
Encelia	palmeri	27.7	-113.6	76	10/30/75	1975	summer	-25.5	331.2	-7.25	0.611	80.5	10.2	1.0	98	2	6	11.56	28.71	16.35	33.28	24.82	1.01	1.12
Encelia	palmeri	27.5	-113.4	46	10/17/74	1974	summer	-27.0	330.3	-7.22	0.682	65.6	12.2	1.8	100	8	30	11.74	29.11	16.01	33.88	24.94	1.06	1.17
Encelia	palmeri	24.1	-110.9	60	10/19/74	1974	summer	-27.5	330.3	-7.22	0.701	61.8	11.4	1.2	103	12	46	13.96	27.58	18.54	30.53	24.54	0.93	0.96
Encelia	palmeri	24.1	-110.9	6	10/31/75	1975	summer	-28.4	331.2	-7.25	0.743	53.2	7.8	1.4	103	12	50	13.96	27.58	18.04	30.79	24.41	0.93	0.99
Encelia	palmeri	24.1	-110.9	6	10/31/75	1975	summer	-26.9	331.2	-7.25	0.676	67.0	5.1	0.9	103	12	50	13.96	27.58	18.04	30.79	24.41	0.93	0.99
Encelia	palmeri	24.1	-110.9	15	2/28/78	1978	spring	-27.5	335.5	-7.34	0.697	63.5	9.9	4.2	103	1	5	13.96	27.58	11.75	25.41	18.58	0.93	0.81
Encelia	palmeri	24.1	-110.9	15	2/28/78	1978	spring	-24.5	335.5	-7.34	0.564	91.4	10.3	5.1	103	1	5	13.96	27.58	11.75	25.41	18.58	0.93	0.81
Encelia	palmeri	24.1	-110.9	15	2/28/78	1978	spring	-25.7	335.5	-7.34	0.616	80.5	10.8	4.5	103	1	5	13.96	27.58	11.75	25.41	18.58	0.93	0.81
Encelia	virginensis	36.3	-116.8	792	2/7/78	1978	spring	-27.1	335.5	-7.34	0.682	66.8	7.4	3.2	57	17	69	15.63	31.59	8.68	23.07	15.88	2.83	1.19
Encelia	virginensis	36.9	-117.3	457	4/2/75	1975	spring	-26.8	331.2	-7.25	0.670	68.2	4.3	3.0	79	6	26	10.55	27.34	2.07	17.17	9.62	2.07	0.80
Encelia	virginensis	36.9	-117.3	457	4/2/75	1975	spring	-26.7	331.2	-7.25	0.666	69.2	1.6	1.3	79	6	26	10.55	27.34	2.07	17.17	9.62	2.07	0.80
Encelia	virginensis	35.8	-116.2	640	3/18/78	1978	spring	-27.4	335.5	-7.34	0.693	64.3	3.2	2.8	87	21	84	11.92	28.19	5.63	19.99	12.81	2.27	1.00
Encelia	virginensis	36.5	-116.5	823	4/2/75	1975	spring	-26.8	331.2	-7.25	0.670	68.3	6.7	3.1	101	7	29	9.29	25.42	0.82	15.70	8.26	1.87	0.75
Encelia	virginensis	36.8	-114.2	670	11/4/77	1977	summer	-28.3	334.1	-7.31	0.735	55.4	7.2	1.5	145	11	43	11.31	26.95	19.99	36.79	28.39	2.14	3.41
Encelia	virginensis	36.8	-115.3	488	3/15/78	1978	spring	-27.5	335.5	-7.34	0.695	63.9	3.6	2.8	203	37	150	5.34	20.98	0.23	13.42	6.83	1.44	0.61
Encelia	virginensis	36.8	-115.3	488	3/15/78	1978	spring	-27.7	335.5	-7.34	0.707	61.5	4.1	2.3	203	37	150	5.34	20.98	0.23	13.42	6.83	1.44	0.61
Encelia	virginensis	36.8	-115.3	488	3/15/78	1978	spring	-26.4	335.5	-7.34	0.651	73.2	3.9	2.5	203	37	150	5.34	20.98	0.23	13.42	6.83	1.44	0.61
Encelia	virginensis	34	-116.6	800	10/4/77	1977	summer	-28.9	334.1	-7.31	0.759	50.2	2.7	2.9	209	27	108	10.98	27.96	17.89	36.13	27.01	1.94	2.88
Encelia	virginensis	34	-116.6	800	10/4/77	1977	summer	-28.9	334.1	-7.31	0.762	49.7	1.4	2.6	209	27	108	10.98	27.96	17.89	36.13	27.01	1.94	2.88
Encelia	virginensis	35.5	-115.2	1432	10/27/74	1974	summer	-26.9	330.3	-7.22	0.675	67.1	4.8	1.8	212	26	105	9.19	22.19	17.22	31.07	24.14	1.66	2.60
Encelia	virginensis	36.8	-113.9	823	11/4/77	1977	summer	-25.7	334.1	-7.31	0.621	79.1	2.2	1.3	247	17	68	8.12	23.81	16.77	33.70	25.23	1.74	2.81
